# Monitoring of malaria parasite resistance to chloroquine and sulphadoxine-pyrimethamine in the Solomon Islands by DNA microarray technology

**DOI:** 10.1186/1475-2875-9-270

**Published:** 2010-10-06

**Authors:** Marie Ballif, Jeffrey Hii, Jutta Marfurt, Andreas Crameri, Adam Fafale, Ingrid Felger, Hans-Peter Beck, Blaise Genton

**Affiliations:** 1 Swiss Tropical and Public Health Institute, Department of Medical Parasitology and Biology of Infection, Department of Epidemiology and Public Health, Socinstrasse 57, 4002 Basel, Switzerland; 2 University of Basel, Petersplatz 1, 4003 Basel, Switzerland; 3 Country Liaison Office, World Health Organization, Honiara, Solomon Islands; 4 National Vector Borne Diseases Control Programme, Ministry of Health and Medical Services, Honiara, Solomon Islands; 5 Department of Ambulatory Care and Community Medicine, Infectious Disease Service, University Hospital, Lausanne, Switzerland

## Abstract

**Background:**

Little information is available on resistance to anti-malarial drugs in the Solomon Islands (SI). The analysis of single nucleotide polymorphisms (SNPs) in drug resistance associated parasite genes is a potential alternative to classical time- and resource-consuming *in vivo *studies to monitor drug resistance. Mutations in *pfmdr1 *and *pfcrt *were shown to indicate chloroquine (CQ) resistance, mutations in *pfdhfr *and *pfdhps *indicate sulphadoxine-pyrimethamine (SP) resistance, and mutations in *pfATPase6 *indicate resistance to artemisinin derivatives.

**Methods:**

The relationship between the rate of treatment failure among 25 symptomatic *Plasmodium falciparum*-infected patients presenting at the clinic and the pattern of resistance-associated SNPs in *P. falciparum *infecting 76 asymptomatic individuals from the surrounding population was investigated. The study was conducted in the SI in 2004. Patients presenting at a local clinic with microscopically confirmed *P. falciparum *malaria were recruited and treated with CQ+SP. Rates of treatment failure were estimated during a 28-day follow-up period. In parallel, a DNA microarray technology was used to analyse mutations associated with CQ, SP, and artemisinin derivative resistance among samples from the asymptomatic community. Mutation and haplotype frequencies were determined, as well as the multiplicity of infection.

**Results:**

The *in vivo *study showed an efficacy of 88% for CQ+SP to treat *P. falciparum *infections. DNA microarray analyses indicated a low diversity in the parasite population with one major haplotype present in 98.7% of the cases. It was composed of fixed mutations at position 86 in *pfmdr1*, positions 72, 75, 76, 220, 326 and 356 in *pfcrt*, and positions 59 and 108 in *pfdhfr*. No mutation was observed in *pfdhps *or in *pfATPase6*. The mean multiplicity of infection was 1.39.

**Conclusion:**

This work provides the first insight into drug resistance markers of *P. falciparum *in the SI. The obtained results indicated the presence of a very homogenous *P. falciparum *population circulating in the community. Although CQ+SP could still clear most infections, seven fixed mutations associated with CQ resistance and two fixed mutations related to SP resistance were observed. Whether the absence of mutations in *pfATPase6 *indicates the efficacy of artemisinin derivatives remains to be proven.

## Background

Shrinking the malaria map is now proposed as a realistic goal in many countries [1,2]. The Solomon Islands (SI) are one of the so defined "elimination countries". Being an insular nation with varying levels of endemicity and transmission on most populated islands [3], and located at the margins of the endemic zones makes it feasible to embark upon malaria elimination. However, to reach this target, it is of crucial importance to gather information about the local malaria epidemiology. Limited information is available on the prevalence of mutations associated with drug resistance or on the population structure of malaria parasites in the SI. The present study provides baseline data on *Plasmodium falciparum *malaria gathered in the Guadalcanal Province in 2004, with focus on anti-malarial resistance.

*In vivo *studies are still considered the gold standard for the assessment of anti-malarial drug efficacy. The analysis of parasite molecular markers has been proposed as an alternative approach for the estimation of resistance to treatment. It has been shown that chloroquine (CQ) resistance is associated with mutations in *pfmdr1 *(*P. falciparum *multidrug resistance gene, coding for P-glycoprotein homologue-1, *pgh1*) and *pfcrt *(*P. falciparum *chloroquine resistance transporter gene) [4-9]. Similarly, it is known that sulphadoxine-pyrimethamine (SP) resistance is associated with mutations in the genes *pfdhfr *(dihydrofolate reductase) and *pfdhps *(dihydropteroate synthase) [10-14]. Mutations in *pfATPase6 *have been described as potential molecular markers involved in resistance to artemisinin derivatives [15]. However, solid evidence for their association with artemisinin resistance is lacking to date.

The analysis of such parasite mutations has the advantage over classical *in vivo *studies that it can be conducted on samples collected on the first day of health centre attendance. It is therefore independent of compliance and circumvents the need for time- and resource-consuming follow-up, which often leads to significant patient loss.

Most of the previous studies on parasite mutations were based on samples obtained from patients prior to treatment. However, there is evidence that the molecular profile of parasites circulating in the community matches the one observed among patients attending health centres. Talisuna *et al *[16] showed that the presence of *pfdhfr *mutations in the community was independently correlated with the clinical treatment outcome. Therefore, not only symptomatic patients, but also asymptomatic individuals could be used to establish the prevalence of single nucleotide polymorphisms (SNPs) in the parasite population [17].

The classical molecular techniques are based on individual examination of SNPs by restriction fragment length polymorphism (RFLP) analysis, allele-specific amplification or DNA sequencing. Unfortunately, these methods are labour-intensive if performed on many SNPs, especially for the analysis of large sample sizes. The analysis of multiple SNPs has been shown to allow a better prediction of the clinical outcome compared to the evaluation of individual SNPs. This argued for a technique facilitating parallel typing of multiple SNPs [9,18-24]. To enable high throughput analysis of several point mutations, a sensitive and rapid SNP typing technique based on DNA microarray was used for the simultaneous assessment of all known resistance-associated SNPs located in genes *pfcrt*, *pfmdr1*, *pfdhfr*, *pfdhps *and *pfATPase6 *[25].

Prior to this study, the malaria drug resistance microarray was validated with field studies conducted in different epidemiological and geographical settings, including Papua New Guinea (PNG) [17,24], and Tanzania. The relationship between SNP prevalence in samples collected in the asymptomatic community and the rate of treatment failure derived from clinical *in vivo *studies was investigated. In PNG, two investigated sites had different rates of treatment failure, which were reflected by different patterns of resistance markers in the corresponding parasite populations [17]. In the frame of that multisite study, the present work reports on an additional field study conducted in 2004 at the north of Guadalcanal province in the SI.

A classical *in vivo *study was conducted to assess the level of CQ+SP failure among symptomatic *P. falciparum *malaria patients. In parallel, a cross-sectional survey in the surrounding asymptomatic community allowed the assessment of SNP prevalence. After the completion of this study, the SI Ministry of Health modified the national treatment guidelines for *P. falciparum *infections. Since 2008, standard first-line treatment for *P. falciparum *malaria has been changed to the combination of artemether plus lumefantrine.

## Methods

### Study area

The study was conducted in Tetere area, Guadalcanal Province of SI between November 2004 and May 2005, roughly corresponding to the rainy season. The SI are located northeast of Australia, at a latitude of 8° South and a longitude of 159° East. The drug efficacy *in vivo *study was conducted at the Lunga clinic, located about 10 km east from Honiara, the capital city of the country. The community-based cross-sectional survey was carried out in 3 villages nearby, which are part of the Lunga clinic catchment area. According to the prevalence of malaria among children aged between 2 and 9 years, the endemicity of *P. falciparum *and *Plasmodium vivax *malaria in that area is considered mesoendemic.

### *In vivo *drug efficacy study

Procedures followed the World Health Organization protocol for 28-day assessment of treatment efficacy for uncomplicated *P. falciparum *malaria in areas of low to moderate transmission [26]. Clinically manifest and microscopically confirmed malaria patients visiting the Lunga clinic were recruited if they were aged > 6 months, had an axillary temperature ≥ 37.5°C or history of fever during the last 48 hours, a *P. falciparum *density between 1,000 and 100,000 asexual parasites per microlitre blood, no signs of severe or complicated malaria and no signs of any other disease. The patients received the first dose of medicine on the day of enrolment (i.e. day 0). Follow-up visits were scheduled on days 1, 2, 3, 7, 14, 21 and 28. Patients were advised to come to the clinic on any other day if symptoms occurred. On every visit, patients were clinically examined and blood samples were taken by finger prick, except on day 1. The parasite density was assessed by microscopy after Giemsa staining of blood slides. In case of a microscopically confirmed *P. falciparum *infection on day 0, standard treatment, which at that time was CQ+SP, was administered under supervision. CQ was given on days 0, 1 and 2 (10 mg/kg chloroquine phosphate per day) and SP was given as a single dose on day 0 (25 mg/kg sulphadoxine + 1.25 mg/kg pyrimethamine). Patients failing first-line treatment were treated with quinine (10 mg/kg quinine sulphate, three times a day for three days) plus a single dose of SP (25 mg/kg sulphadoxine + 1.25 mg/kg pyrimethamine, on the first day of second-line treatment).

### Community-based cross-sectional survey

Three villages within the catchment area of the clinic have been selected for the community-based cross-sectional survey. Clinical symptoms during the last seven days, history of malaria and consumption of anti-malarials were reported on an individual questionnaire. Axillary temperature was taken with a digital thermometer and blood samples were collected by finger prick. After microscopy examination of the slides, *P. falciparum *positive people were informed of the result and treated with the first-line treatment at that time.

### Molecular analyses

To distinguish a true recrudescence from a new infection (*in vivo *drug efficacy study), and to determine PCR prevalence of *P. falciparum *infections and the multiplicity of infection (MOI) in the community, paired *in vivo *study and community survey samples were genotyped by PCR-RFLP of *P. falciparum msp2 *(merozoite surface protein 2) [27]. In brief, after DNA extraction (QIAamp^® ^DNA Blood Kit, Qiagen, Switzerland), the *msp2 *gene was amplified by nested PCR and digested with restriction enzymes Hinf I and Dde I. Restriction fragment patterns were analysed after electrophoresis on 10% polyacrylamide gels.

**Figure 1:**
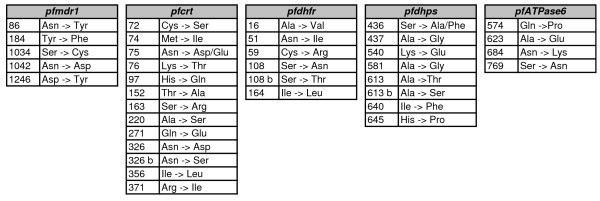
**List of the 34 anti-malarial resistance SNPs assayed on the DNA microarray**. CQ resistance-related markers included 5 SNPs located in *pfmdr1* and 13 SNPs located in *pfcrt*. SP resistance-related markers included 6 SNPs located in *pfdhfr* and 6 SNPs located in *pfdhps*. Putative artemisinin-resistance related markers included 4 SNPs located in *pfATPase6*.

Mutations associated with anti-malarial resistance were assessed by microarray technology among *P. falciparum *positive samples as described in detail elsewhere [25]. The method is based on the parallel PCR amplifications of the target sequences followed by primer extension using fluorochrome-labelled ddNTPs. After denaturation, primer extension products were specifically hybridized onto the microarray. All SNPs assessed on the microarray are listed in Figure 1.

The data output given by the Axon GenePix™software (Molecular Devices Inc., USA) was edited and converted into a table listing all SNPs with their respective genotype (i.e., wild-type, mutated or mixed) for each patient. For quality control of the SNP microarray typing, data of some of the samples were confirmed by sequencing (Macrogen Inc., Korea).

### *In vivo *drug efficacy

According to the microscopic observation of patient blood smears and after PCR correction by *msp2 *genotyping, the individual treatment outcomes were classified according to the categories defined by WHO for low to moderate transmission areas [26]: Early Treatment Failure (ETF), Late Clinical Failure (LCF), Late Parasitological Failure (LPF), and Adequate Clinical and Parasitological Response (ACPR). The treatment failure rate among *P. falciparum *malaria patients was calculated for patients who successfully completed the study. The overall treatment failure (TF) rate was obtained by pooling ETF, LCF and LPF rates.

### Ethical approval

The National Ethics Committee of the SI and the Basel Ethic Commission (Ethikkommission beider Basel) in Switzerland reviewed and approved the study protocol. All communities and patients (or parents and legal guardians) involved were enrolled in the study after having given informed consent.

## Results

The field procedures were conducted in 2004-2005 in the North coast of Guadalcanal Province in the SI.

### *In vivo *study

A total of 43 (100%) patients aged between 2 and 23 years (median: 9 years) completed the *in vivo *study. No patient was lost to follow-up. 18 (41.9%) patients were included without meeting the required criteria (i.e. they were without fever (or history of fever) and/or had a parasite density below 1,000 *P. falciparum*/μl on day 0). These individuals were censored from further analysis. Among the 25 (58.1%) remaining patients the following outcomes were observed after PCR correction: 22 (88%) ACPR, 1 ETF (4%) and 2 LPF (8%), giving a total of 12% TF.

### Community-based cross-sectional survey

Three villages comprising the catchment area of the Lunga clinic were screened for asymptomatic *P. falciparum *infected individuals. Due to very limited census data from those three villages, the combined total population could only be roughly estimated to 1600 inhabitants. A total of 388 asymptomatic individuals (24.3% of the estimated total population) aged between 1 and 73 years (median: 17 years) were screened in the community, among them 53.4% females. Based on microscopy reading of blood smears, 50/388 (12.9%) individuals were infected with *P. falciparum*; using *msp2 *PCR, 97/388 individuals were found positive, giving a prevalence rate of 25% *P. falciparum *malaria.

RFLP analysis was successfully conducted on 80 samples. The *msp2 *PCR fragment revealed 56 (70%) single infections, 18 (22.5%) double infections, five (6.25%) triple infections and one (1.25%) quadruple infection. The mean multiplicity of infection (MOI; mean number of co-infecting parasite clones) in *P. falciparum *positive samples was 1.39.

Ten PCR reactions were performed for the five genes associated with drug resistance. Of the 97 *P. falciparum *positive samples analysed, 21 (21.6%) showed poor amplification for the majority of the PCR reactions (> 5 poor PCR results out of 10) and had thus to be excluded. The 76 (78.4%) remaining samples were used for genotyping by microarray. Sixteen unclear microarray typing outcomes were confirmed by sequencing of the corresponding gene fragments (Macrogen Inc., Korea).

**Figure 2:**
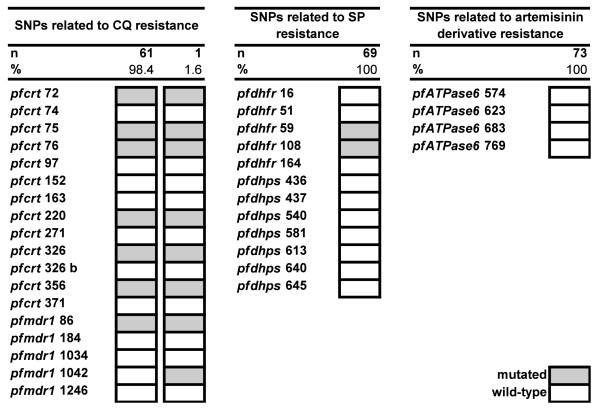
**Observed haplotypes of SNPs related to resistance to CQ, SP and artemisinin derivatives**. A haplotype is defined by the combination of mutations in genes related to drug resistance. Grey indicates mutations and white indicates wild-type. Two different haplotypes were observed in genes *pfmdr1 *and *pfcrt*. One haplotype was observed in genes *pfdhfr *and *pfdhps*. One haplotype was observed in gene *pfATPase6*. n corresponds to the number of samples assessed for the analysis.

Overall, the analysed population showed to be very homogenous (Figure 2). Two different haplotypes were observed for the two CQ resistance-associated genes. With 70% of the patients having a single infection and the unambiguous DNA microarray signals, it was considered that co-infecting clones had the same pattern of SNPs. The most dominant haplotype showed mutations at positions *pfmdr1 *N86Y and *pfcrt *C72 S, N75D/E, K76T, A220 S, N326 D, and I356L, and was observed in 98.4% (61/62) of the population. The second haplotype was observed in one sample only (1.6%) and differed from the first one by having one additional mutation at position N1042 D in *pfmdr1*. Other SNPs related to CQ resistance were all wild-type.

For the genes involved in SP resistance, mutations were fixed at positions C59R and S108N in *pfdhfr*. Other SNPs in *pfdhfr *and *pfdhps *were all wild-type. No mutation was detected in *pfATPase6*.

## Discussion

The outcome of CQ+SP against falciparum malaria was investigated at a clinic located close to Honiara in the SI. At the same time, the presence of resistance-associated SNPs in *P. falciparum *was assessed in the surrounding asymptomatic community, screened by a cross-sectional survey.

The resistance-associated SNPs observed in the sample set are in line with previous findings from other malaria areas worldwide. Regarding SP resistance, all samples presented two fixed mutations at positions C59R and S108N in *pfdhfr *and none in *pfdhps*. The importance of cumulative mutations conferring SP resistance has been described; in particular the combination of the SNPs *pfdhfr *S108N, N51I, C59R and *pfdhps *A437G, K540E [9,28] being correlated with anti-folate treatment failure. The double mutant *pfdhfr *C59R+S108N, previously shown to be associated with treatment failure [23], has been reported at high prevalence in the SI and other Melanesian countries before [11,29,30]. Regarding CQ resistance, fixed mutations at positions *pfcrt *C72 S, N75D/E, K76T, A220 S, N326 D, I356L and *pfmdr1 *N86Y were observed. The crucial role of *pfcrt *K76T in conferring CQ resistance is well known, together with additional mutations in *pfcrt *maintaining functional properties [5,31]. The mutation *pfmdr1 *N86Y is considered as a modulator of CQ resistance in the presence of *pfcrt *mutations [32-34].

The present study was part of a multisite project simultaneously conducted in Tanzania (Mugittu *et al*, unpublished) and PNG [17,24]. In this context, it is interesting to compare the observations from the SI with reports from PNG due to the close location of both countries (PNG is situated about 1500 km West of Honiara) and the common origin of the mainly Melanesian human population. The SNPs seen in PNG community samples in 2003-2005 reflected the SI observations with high mutation frequencies in *pfcrt *and *pfmdr1*, moderate mutation frequencies in *pfdhfr*, and rare mutations in *pfdhps *[24]. Also similar to the SI, the mutation *pfmdr1 *N1042 D was only seen in 1% of the PNG patients. Mutations in *pfATPase6 *were neither observed in the SI, nor in PNG. The observation of *pfATPase6 *mutations in French Guiana has so far not been confirmed elsewhere [35,36].

Due to the small sample size, conclusions on the correlation between the prevalence of molecular markers in the parasite population infecting the community and the *in vivo *treatment failure rate cannot be drawn. The *in vivo *study conducted at the clinic showed 12% of CQ+SP failure, which can partially be explained by the fixation of the SP resistance-conferring *pfdhfr *C59R+S108N double mutant in the parasite population. However, PNG data showed that not all mutations predict treatment failure at the same level [24]: mutations *pfmdr1 *N86Y and *pfdhps *A437G were the strongest predictors of amodiaquine+SP failure. It can be postulated that the absence of the *pfdhps *A437G mutation explains the lower rate of treatment failures in the SI compared to PNG and indicates that SP, and even more specifically sulphadoxine, might have been the effective component of the drug combination. The poor efficacy of CQ has indeed already been reported in the 1980's, when CQ monotherapy was used (Bobogare *et al*., unpublished data). The combination of CQ+SP used in the 1990 s as second-line treatment, and subsequently as first-line treatment between 2003 and 2007, certainly further maintained a CQ pressure and thus its limited efficacy.

The use of DNA microarray allowing the parallel analysis of antimalaria drug resistance markers was particularly advantageous in the context of CQ+SP as first line treatment, since many polymorphisms have been described for those two drugs. In contrast, no marker is validated as indicators of artemether plus lumefantrine combination therapy, which has replaced CQ+SP in the SI since 2008. The determination of the *pfmdr1 *copy number in samples collected after 2008 could provide a useful complement for the assessment of potential resistance to artemether and lumefantrine.

The observed low genetic diversity of the *P. falciparum *population does probably reflect the context of an island country, where gene flow may be restricted. This low diversity was compared with observations in PNG [24]. In contrast to the SI situation, where mutations associated with antimalarial drug resistance tended to be fixed, the diversity was higher in PNG with various SNP patterns present in the *P. falciparum *population. However, the most dominant SNP pattern related to CQ resistance seen in PNG between 1992 and 2002 [37] was composed of mutations *pfcrt *K76T, A220 S, N326 D and I356L, which corresponds to the dominant haplotype observed in the SI. Regarding SP resistance, the dominant SNP pattern in PNG was the *pfdhfr *C59R+S108N double mutant, which also corresponds to the dominant haplotype in SI.

Parasite populations in SI and PNG show similarities that can be explained by their close geographical vicinity. However, the pronounced insularity and remoteness of the SI differs from PNG and this may be reflected in the lower genetic diversity observed in the SI. The absence of mutations in *pfdhps *in the SI, which are already present in PNG, is a possible illustration of such low gene flow. A meta-population analysis on the diversity of genes encoding *P. falciparum *vaccine antigens also reported that biogeographic characteristics of island nations in the Pacific constitute a barrier to gene flow [38]. Restricted gene flow is also reflected in the low mean multiplicity of infection in the SI (1.39 concurrent infections in parasite positive samples). For comparison, in a coastal location in PNG having similar geographical characteristics, the multiplicity of infection was slightly higher (1.54), and the *msp2 *PCR-based *P. falciparum *prevalence was also higher than in the SI (32% versus 25%, respectively) [39].

An additional explanation for limited diversity in the considered study site could be the vector control measures. Field evaluations have shown that insecticide-impregnated bed nets and indoor residual spraying together with educational activities significantly reduced malaria transmission in north Guadalcanal Province and contributed to a slow decrease of malaria incidence in the SI since 1992 [40].

To further validate the hypothesis of restricted gene flow in the SI in contrast to PNG, the diversity of various microsatellites which are not under selection pressure could be tested.

In conclusion, the present data provide baseline information on the prevalence of *P. falciparum *drug resistance-associated SNPs and highlight the low level of genetic diversity in the *P. falciparum *population in the Guadalcanal Province of the SI. Surveillance of molecular markers of drug resistance should be an integral part of the planned malaria eradication programmes, which are currently initiated in insular settings, so that the resistance dynamics can be assessed and the most effective treatment selected.

## Competing interests

The authors declare that they have no competing interests.

## Authors' contributions

MB participated in the field work, conducted the laboratory-based analyses and wrote the manuscript; JH participated in the study design and field work coordination and edited the manuscript; JM contributed to the molecular analyses and edited the manuscript; AC supervised the microarray analyses; AF participated in the field work; IF helped to draft the manuscript; HPB participated in the study design, supervised the laboratory work and helped to draft the manuscript; BG conceived the study, participated in its design and coordination, and helped to draft the manuscript. All authors read and approved the final manuscript.
